# Ten Simple Rules on How to Organize a Scientific Retreat

**DOI:** 10.1371/journal.pcbi.1005344

**Published:** 2017-02-02

**Authors:** Julia Ponomarenko, Romina Garrido, Roderic Guigó

**Affiliations:** 1 Centre for Genomic Regulation (CRG), Barcelona Institute of Science and Technology, Barcelona, Catalonia, Spain; 2 Universitat Pompeu Fabra (UPF), Barcelona, Catalonia, Spain; 3 Institut Hospital del Mar d’Investigacions Mediques (IMIM), Barcelona, Catalonia, Spain

## Introduction

Scientific retreats are an intrinsic part of the life of many institutes, departments, and groups. They depart from traditional, virtual [1], and unconventional conferences [2], workshops [3], and other types of scientific meetings [4] in that participants generally all know each other prior to the retreat, and, often, they have a good grasp of the scientific interests and accomplishments of each other; they may even be working closely together. Participants, thus, do not attend the retreat expecting to necessarily hear about breakthroughs in their fields of interests or to present their latest results to an expert audience but rather to have a deeper knowledge of the work of their closest colleagues, learn from developments in related areas, and explore potential collaborations.

Since retreats usually take place away from the home institute and may expand over two or more days, they are expensive to organize—including significant institutional funds and employees’ working and personal time. They are disruptive of the daily scientific routine: experiments may need to be stopped or planned ahead, and regular activities such as seminars and group meetings need to be cancelled. Thus, to many, the benefits of moving a group of people who already share the same working space to a remote location over a period of two or more days are not obvious. After all, retreat participants already have the opportunity of meeting, almost on a daily basis, at the home institute.

There is little empirical evidence that scientific retreats lead to better science (whatever this exactly means); we have been unable to find any work that would correlate frequency or length of scientific retreats with any of the metrics usually employed to measure the quality of science. Yet, anecdotal evidence of a positive correlation between scientific breakthroughs and scientists being outside the lab is abundant and includes a discovery of penicillin attributed to a long summer vacation by Fleming in 1928 [5] or a discovery of Velcro by Georges de Mestral after a hunting trip with his dog in 1941 [6]. More recently, the invention of a new cipher for using DNA as high-capacity data storage by Ewan Birney and Nick Goldman of the European Bioinformatics Institute (EBI) apparently happened involving “many beers” [7, 8].

If properly planned, retreats offer an informal environment, which is becoming increasingly rare with the “laborization” of science, when scientists tend to follow preestablished working schedules and interact with each other only during the regular working hours, following well-structured formats of group meetings, conference calls, seminars, and other meetings. There is also an increasing divide between work at the lab and personal life. These tendencies are new to science, often being seen in the past as a way of life rather than a means of living. Retreats offer the possibility to break these tendencies—even if only for a short period of time—by bringing together work and personal life. At the retreat, a student may have a lunch with a professor he never had a chance to interact with, postdocs from different groups may hike and party together, and principal investigators (PIs) with little time for informal discussions during a normal working week may have a chance to debate about their favorite topics and get to know each other. All these apparently irrelevant events may result in productive science. Smaller retreats—involving, for example, a single research group—can provide an opportunity for improving the day-to-day group dynamics [9] and boosting creativity [10]. Retreats can also facilitate building trust amongst people that is vital for productive working relationships [11]. The authors believe that the intangible benefits delivered by scientific retreats are currently vastly underrated and understudied.

Retreats may come in many flavors. They may appeal to a particular constituency (PhD students, postdocs, PIs), and they may involve hundreds of participants from an entire institute or barely a dozen from a single research group. They may be attended only by scientists or they may also include administrative staff. For the past 11 years, the authors have organized a yearly two-day retreat for the Bioinformatics and Genomics Program at the Center for Genome Regulation (CRG) in Barcelona, Catalonia. This event also includes computational biology groups from other programs at CRG as well as other institutions from the Barcelona Biomedical Research Park (PRBB). It could be described as a departmental retreat, attended by students, postdocs, PIs, technicians, and administrative staff. The 2016 retreat took place in a rural mountain hotel an hour’s driving distance from Barcelona. In previous years, we opted for a beach location or a ski resort, always no more than two hours from the city. In 2016, there were more than 110 scientists present, the majority of whom were PhD students and postdocs. According to the feedback we have received from the participants, the retreat provided a memorable and for some participants even a life-changing experience.

With scarcity of information on how to organize a scientific retreat, organizers often opt to follow a standard formula of a conference with an extensive scientific program. And while retreats and other scientific events share common rules, many of which can be found in the *PLOS Computational Biology* “Ten Simple Rules” collection [1–4], here we emphasize rules applied specifically to retreats: in particular, departmental-style retreats.

## Rule 1: Define the Purpose

Decide first on why (and whether) you need the retreat. Engage participants in deciding about the purpose and clearly communicate the purpose to participants. Depending on the type of the retreat and the participants, the answers might be found in the following statements: “Discuss the future of the field,” “Discuss career perspectives,” “Learn what colleagues are working on,” “Get to know your colleagues,” “Foster the feeling of belonging and bonding,” “Make friends,” and “Relax and have fun.” A pre-retreat survey can help to learn about the participants’ expectations and needs. Our latest pre-retreat survey is provided in S1 Text.

By launching a pre-retreat survey, which was answered by 99 participants of the previous retreat, we learned that we should focus on the three major goals: “Exchange information on who is working on what with the goal to foster new collaborations,” “Get to know each other better,” and “Have fun.” Those goals were logical for our retreat because it gathered scientists from five institutions at the PRBB campus, many of whom were students and postdocs of multiple affiliations. In the end, we defined the goal of the retreat as “Fostering the feeling of belonging in our shared computational biology community.”

## Rule 2: Define the Budget, Length, and Time of the Retreat

The length and time of the retreat are substantially defined by the available budget.

Although a two-day retreat might be of the ideal length—since spending one night out gives much more opportunities for interaction—if the budget is limited and the number of participants is small, a one-day retreat can be justifiable, with dinner or just going out to the bar at the end of the day. A retreat is an intense event and extending it to more than two days may be exhausting.

It should be expected that the retreat cost can considerably increase at summer time, school breaks, and holiday seasons; also, depending on personal circumstances, it may be complicated to organize it during a weekend. Therefore, we recommend organizing a retreat at the beginning or at the end of a working week. Considering numerous events happening during a year (symposiums, training, classes, attestations, seminars, thesis defenses, etc.), it is extremely important to schedule a retreat a year in advance.

From our experience, we suggest budgeting or taking care of the following on your own:

- transportation to and from the venue by bus, boat, train, etc.
- lodging
- meals: breakfasts, coffee breaks, lunches, dinner(s)
- meeting rooms and equipment (microphones, projectors, computers, boards, laser pointers, etc.)
- name and/or team badges or stickers
- equipment and/or help for outdoor activities (e.g., volleyball equipment, local hiking guide, etc.)
- room and/or place for a party and equipment (karaoke, music, projector, etc.)
- music playlist or a DJ
- bar (define and communicate drinking policies in advance: who pays for drinks, how much they cost, open and closing times of the bar, etc.)

## Rule 3: Decide on the Venue

Depending on the retreat goals, budget, weather, and the number, age, and seniority of participants, the venue has to be carefully selected to provide opportunities for all planned activities and productive encounters. The venue is hugely important for the retreat success since, in contrast to a conference, people cannot escape. They have to be physically present for the whole duration of the event in the same environment where they will be sleeping, eating, resting, talking, drinking, etc.

For the most recent retreat, we selected an isolated rural hotel that provided two large conference rooms, a large dining room, various cozy and private places for encounters and discussions, as well as plenty of opportunities for outdoor activities, such as hiking, football, an adventure park, and a swimming pool. There was also an indoor entertainment hall with a bar for parties, dancing, and karaoke.

Transportation to and from the venue needs to also be considered. As a rule, for each retreat, CRG provides a coach from and to the work place. But since some participants live in different parts of and outside Barcelona, upon registration we ask participants if they will be using a coach or not. Before the retreat, the lists of those who will use a coach are made for checking in everyone on the way to the bus. A car-sharing signup option can also be considered.

## Rule 4: Establish Policies

It is your responsibility as a retreat organizer to ensure the participants’ safety and appropriate behavior during the retreat to avoid any kind of harassment, including sexual [12]. If your organization has policies written specifically for retreats and/or general rules of conduct, we suggest reminding participants about these policies at the time of registration, emphasizing that the retreat is an extension of a work environment and all rules of conduct have to be respected. However, if there are no such policies in place or they need to be changed, use the upcoming retreat as an opportunity to address this issue.

Depending on the local customs and law, you might consider outlining media policies regarding using mobile phones, taking pictures, and recording during the retreat. To promote the feeling of trust and to allow people to relax, you might also decide on a rule about sharing the retreat collaterals (see Rule 10).

Do not overlook the gender balance issue [13], considering both the scientific and social programs and while forming the retreat committees.

## Rule 5: Set Up the Agenda and Appoint Committees with Responsibilities and Timelines

The program should include time for scientific and team-building activities, food and entertainment blocks, and plenty of free time. Generous timing should also account for any unforeseen issues and hiccups, such as bad weather, talk cancellations, broken equipment, etc. Do not also overlook local customs concerning meal and bedtime; for example, in Spain, lunch at very early afternoon hours, dinner before 8–9 pm, or a party stopped at midnight would be considered unusual. The agenda of our 2016 retreat is provided in S2 Text.

We can recommend setting up an organizing committee including one secretary or administrator and one or two senior scientists or professors. The organizing committee should take care of the logistics, finances, and general coordination of the retreat. Our experience also shows that at least two additional committees are helpful to form: a scientific committee that will take care of the agenda, scientific sessions, and other scientific events, and a social committee that will organize social activities.

A kickoff meeting defining the main goals, expectations, responsibilities, and timelines of all committees should be organized at least three months in advance. Afterwards, committees can work independently. The final meeting should take place a few days before the retreat.

## Rule 6: Arrange an Engaging Science Program

It can be regrettable to spend precious retreat time on presentations that can be delivered via seminars, meetings, data clubs, and other regular institutional gatherings. We recommend the retreat for probing unusual formats, some of which have been employed by “unconferences” [9]. Think of, for example, organizing a few topic-centered sessions of short talks delivered by both faculty and students, followed with a panel discussion. Depending on the participants and the size of the retreat, it might be useful to include tutorials, short workshops, and round-table discussions. Debates and games, such as scientific quizzes, scientific “speed dating” [14], and “fishbowl” [15] are excellent and engaging forms to discuss various topics. As quizzes were a staple of previous retreats, this year we decided to organize Oxford-style debates, with two debating teams and a moderator. Run at the climactic time of the retreat before the first day’s dinner, two debates—one discussing what a gene is and the other the cons and pros of pre- versus post-publishing reviews and the future of scientific publications—provided enough fuel for the buzz to continue during dinner.

From our survey, it also became clear that participants wanted to hear from PIs about their careers and about the future of the field. We therefore asked three PIs who are about to finish their junior tenure and move to other institutions to each give an hour talk reflecting on their careers and their views on the science. These talks were engaging and moving for both PIs and audience; they also created an intimate and special atmosphere for the whole retreat. Indeed, it has been shown in multiple studies that memories are more readily made and logical reasoning performance is improved when positive emotions are stirred [16, 17].

## Rule 7: Organize a Range of Social Events

Without outdoor activities or time to relax, nobody would feel that they are at a “retreat.” Do not however rely only on self-organization by just allocating time in the program for these activities. Plan the activities sensibly: for example, if you schedule in the morning of the second day time for volleyball, nobody will show up to play; likewise, free time for hiking after lunch will likely turn out to be a time for a swim or a nap. Instead, explicitly organize every hour of activities, accommodating various interests and physical abilities. The latter cannot be stressed enough as you cannot force everyone to participate in extreme physical activities that may as well involve humiliating moments.

In the pre-retreat survey, we asked people about outdoor and team-building activities they wanted to suggest and would be interested in participating and/or organizing. After selecting a few activities and forming organizing teams, we asked participants to register for as many activities as they wished, specifying a ranking of choice. In the result, we had two hikes of different difficulty levels, a 10-km run competition, a football competition, a capoeira session, and a tree-climbing activity in an adventure park. The majority participated in at least one of these activities, while some still opted for a couch, a swimming pool, or a good read. Most important was that everyone could return from the retreat to work with renewed energy and motivation.

The retreat is not a retreat without a proper planned after-dinner party. Why is partying important? It is time to celebrate excitement for hard work and to rejuvenate the team spirit. It allows people to bond with and to show gratitude towards each other. It fosters camaraderie, since it is easier to see yourself as a team when you are enjoying yourselves together. It allows improving verbal and nonverbal communication skills. To avoid embarrassments and tiredness associated with excessive drinking, do not rely only on a DJ, a bar, and self-organization. Get creative: think about having science-related themes for the party. For some retreats, we organized scientific competitions, games, and quizzes. This year we organized a team karaoke “Eurovision-like” competition with awards and randomly formed teams. We suggest involving participants in the organization of the entertainment. Engage a good dancer to encourage people to dance in formation, which provides a tangible bonding experience. Ensure that the place has proper equipment and space for a party. Have your most party-inclined members take the lead, or, if the budget allows, consider hiring a professional entertainer to help to get the party going after a long retreat day.

## Rule 8: Ensure Everyone Is Included

The retreat is about its participants; and to make it engaging, productive, and memorable for everyone, it must include enough opportunity for each individual to speak and to work on their “soft skills.” Activities organized by committees formed by participants, such as a debate, a game, a sport, or a karaoke competition, give an excellent opportunity for everyone to tap into hidden potential and train hugely important team-building and communication skills.

Diversity of personality types or traits [18, 19] and team roles [20–22] should be also taken into account while planning talks, debates, and other activities so that strengths and competences will be matched to particular roles. For example, you might think whether randomly formed teams will work for a particular activity or if it is valuable to organize teams in advance.

Here is an example of how we used the format of short talks and an informal lunch as team- building and public speaking exercises. Sessions of two-minute lightning talks were regularly run at this retreat and were loved by speakers but not so much by the audience, who were lost in the ocean of talks. This time, we decided to have a single one-hour session of three-minute teaser talks using the format: “The scientific problem that twists my brain and I want to discuss during lunch is…” Individuals were randomly assigned to teams of six. The number of talks corresponded to the number of teams, and each team was assigned a table to discuss the lightning talk during the following lunch break. Knowing about this format in advance, some presenters chose to raise topics requiring brainstorming—for example, “How to start a PhD thesis without getting lost” or “From circular to linear: How to represent gene order?” This approach of discussing science around a very specific problem during lunch proved to be productive and fun.

## Rule 9: Plan Ahead and Keep to Time

There are many large and small tasks to take care of well in advance of the retreat, including registration, pre-retreat surveys, transportation, dietary and/or accessibility requirements, scheduling of each event, name badges, equipment, and many others. If not planned in advance, some things may take an unexpectedly long time and cause a disruption in the schedule: take, for example, the logistics of checking in 100 people at the hotel.

The importance of good time keeping cannot be stressed enough, and importantly, it concerns not only talks and mealtimes but all other events as well. Remember to wrap up the event with a conclusion, accolades, and awards (see Rule 9 of Corpas et al. [4]).

Have an efficient and experienced administrator to manage the retreat logistics. It must be a rule for organizing any event: if you cannot count on an excellent administrator, you are doomed to fail. For more on what an administrator might and should delegate, see Rule 6 of Corpas et al. [4].

The success of the retreat also depends on the level of participants’ involvement and commitment; so ideally, set up a dedicated committee for each program area.

## Rule 10: Gather and Give Feedback

Sharing the retreat collaterals—presentations, photos, and videos—will make it more memorable. At our organization, Dropbox is commonly used to share folders and files; therefore we set up a Dropbox folder shared with all retreat participants. Other organizations might opt for a different platform. What is most important is creating shared memories and experiences that are key factors in team building and producing good leaders in your organization and/or community [23].

Of course, there cannot be a perfect retreat, so gathering feedback on what worked and what did not can help planning for the next time. We usually gather feedback about retreats, courses, and other events via online surveys; in our experience, a survey conducted immediately after an event engages up to 80%–90% participants. We also suggest obtaining feedback on new ideas and proposed format changes a few months before planning the next retreat via a pre-retreat survey.

At the retreat, an administration and group leaders can also learn a lot about their leadership style and which type of leaders they have in their groups [24–26], as well as what kinds of personalities are needed for the group to be more productive, creative, and stress-free [27].

## Supporting Information

S1 Text Pre-retreat survey of 2016. (DOCX)

S2 Text 2016 retreat agenda. (DOCX)
