# Supplementary figures and images for: Ten Simple Rules on How to Organize a Scientific Retreat
Source: PLoS Comput Biol. 2017 Feb 2;13(2):e1005344. doi: 10.1371/journal.pcbi.1005344 (PMC5289491; doi:10.1371/journal.pcbi.1005344)

**
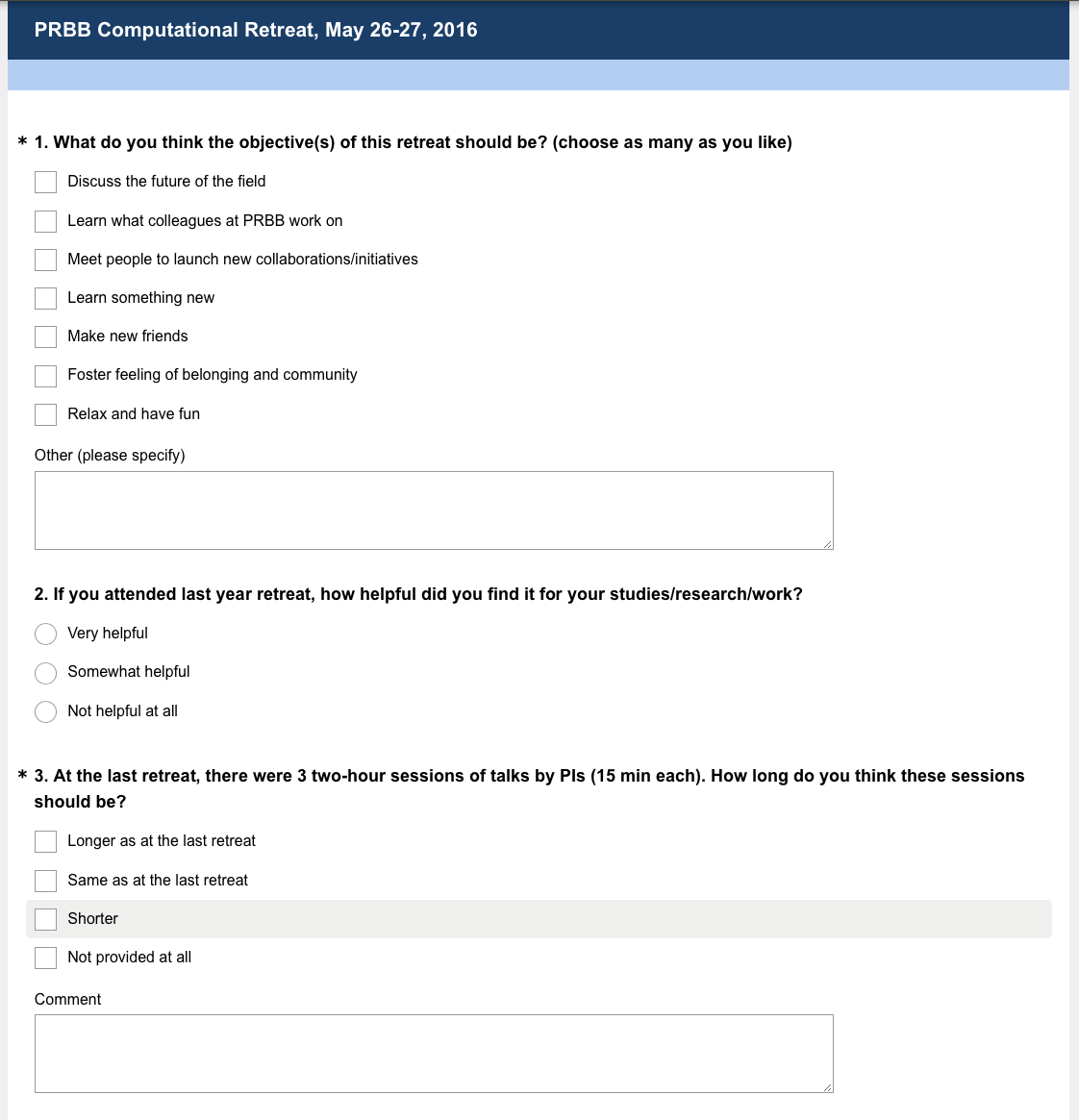
**


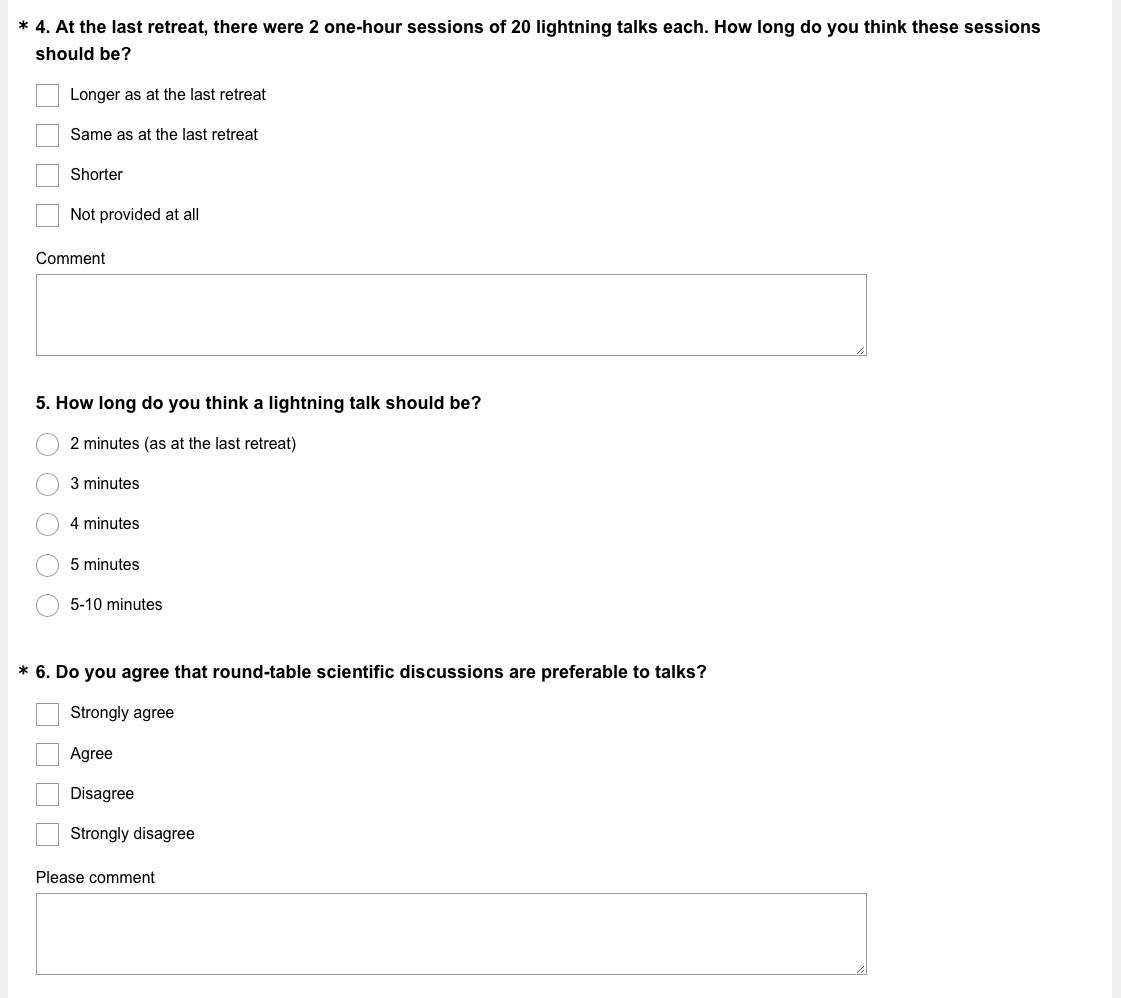


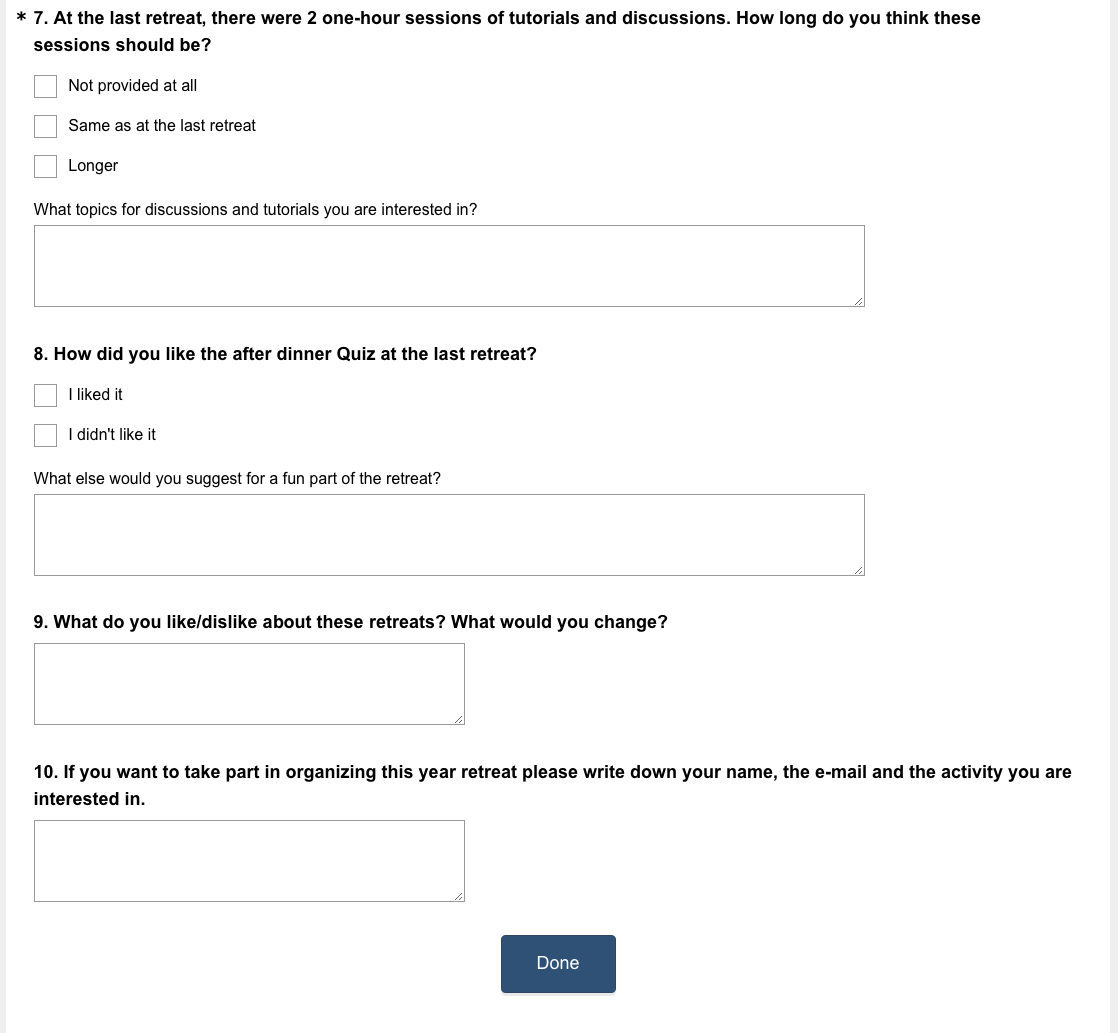

Supplement: S1 Text — (DOCX) [file pcbi.1005344.s001.docx]
