# Supplementary material for: Ten Simple Rules on How to Organize a Scientific Retreat
Source: PLoS Comput Biol. 2017 Feb 2;13(2):e1005344. doi: 10.1371/journal.pcbi.1005344 (PMC5289491; doi:10.1371/journal.pcbi.1005344)
Supplement: S2 Text — (DOCX) [file pcbi.1005344.s002.docx]

**Day 1 - May 26, 2016**

08:00 Meeting at the PRBB ground floor reception

08:15 Departure

*09:45 – 10:30 Coffee and check-in*

10:30 – 10:45 Welcome

**10:45 – 11:45 PI talk (Toni Gabaldon)**

11:45 – 12:45 Lightning talks

*13:00 – 15:00 Lunch*

*14:30 – 15:00 Check-in*

15:00 – 18:30 Hike/Sport/Games

*18:00 – 19:00 Coffee break*

19:00 – 20:00 Debates

*20:00 – 21:30 Dinner*

21:30 – 03:00 Karaoke team competition & Party

**Day 2 – May 27, 2016**

08:00 – 10:00 Breakfast and check-out

**10:00 – 11:00 PI talk (Fyodor Kondrashov)**

*11:00 – 11:30 Coffee break*

11:30 – 13:00 Scientific session

*13:00 – 14:30 Lunch*

**14:30 – 15:30 PI talk (Nuria Lopez)**

15:30 – 15:45 Closing remarks

16:00 Departure

Lightning talks (3 min each)

“*The scientific problem that twists my brain and I want to discuss during the lunch is “*

( Ala ) *Purifying selection acting on tumor genomes.* Luis Zapata (CRG, Ossowski).

( Arg ) *How can we identify significant 3D co-localisation?* Irene Farabella (CNAG/CRG, Martí-Renom).

( Asn ) *Spike-Ins: why, when and how.* Silvia Perez Lluch (CRG, Guigo).

( Asp ) *How to improve CRISPETa?* Estel Aparicio (CRG, Guigo).

( Cys ) *From Data Repository To Knowledge Base -- From Dark Ages To Enlightenment -- Is there a way through?* Catherine Kirsanova (CRG, Ponomarenko).

( Gln ) *From circular to linear: How to represent gene order?* Marina Marcet-Houben (CRG, Gabaldon).

( His ) *What makes a good normalization method (for Hi-C data).* Yannick Gunter Spill (CNAG/CRG, Martí-Renom).

( Ile ) *Why a 21st amino acid?* Didac Santesmasses (CRG, Guigo).

( Lys ) *Nuclear ecology in chimeric syncytia.* Miguel Angel Naranjo (CRG, Gabaldon).

( Met ) *How can I apply recurrent neural networks to predict features of biological sequences?* Stefanie Marti (CRG, Tartaglia).

( Phe ) *Can we use protein structural variations to infer protein evolution?* Cedric Magis (CRG, Notredame).

( Pro ) *Using graph databases as a service.* Toni Hermoso (CRG, Ponomarenko).

( Ser ) *What are the ingredients of a good genome assembly? Let’s share recipes!* Tyler Alioto (CNAG).

( Thr ) *How genomics can improve wildlife conservation practice.* Mateusz Konczal (CRG, Kondrashov).

( Trp ) *Drug prescription data. WWYD?* Kaiser Co (CRG, Guigo).

( Tyr ) *How much chimp are we really?* Lukas Kuderna (UPF, Marques).

( Val ) *How to start my PhD thesis without get lost?* Veronica De Pinho (CRG, Gabaldon).
